# Association of Psychosocial Stress With Risk of Acute Stroke

**DOI:** 10.1001/jamanetworkopen.2022.44836

**Published:** 2022-12-09

**Authors:** Catriona Reddin, Robert Murphy, Graeme J. Hankey, Conor Judge, Denis Xavier, Annika Rosengren, John Ferguson, Alberto Alvarez-Iglesias, Shahram Oveisgharan, Helle K. Iversen, Fernando Lanas, Fawaz Al-Hussein, Anna Członkowska, Aytekin Oguz, Clodagh McDermott, Nana Pogosova, German Málaga, Peter Langhorne, Xingyu Wang, Mohammad Wasay, Salim Yusuf, Martin O’Donnell

**Affiliations:** 1 HRB (Health Research Board) Clinical Research Facility Galway, School of Medicine, University of Galway, Galway, Ireland; 2 Wellcome Trust–HRB, Irish Clinical Academic Training, Dublin, Ireland; 3 School of Medicine and Pharmacology, The University of Western Australia, Perth, Australia; 4 Population Health Research Institute, Hamilton Health Sciences and McMaster University, Hamilton, Ontario, Canada; 5 Division of Clinical Research and Training, St Johns Medical College and Research Institute, Bangalore, India; 6 Cardiology Unit, Sahlgrenska University Hospital/Östra, Gothenburg, Sweden; 7 Department of Molecular and Clinical Medicine, Institute of Medicine, Sahlgrenska Academy, University of Gothenburg, Gothenburg, Sweden; 8 Rush Alzheimer Disease Research Center, Rush University Medical Center, Chicago, Illinois; 9 Department of Neurology, Rigshospitalet, University of Copenhagen, Copenhagen, Denmark; 10 Health and Medical Sciences, University of Copenhagen, Copenhagen, Denmark; 11 Faculty of Medicine, Universidad de La Frontera, Temuco, Chile; 12 Department of Neurology, Faculty of Medicine, King Saud University, Riyadh, Saudi Arabia; 13 Institute of Psychiatry and Neurology, Warsaw, Poland; 14 Department of Internal Medicine, Faculty of Medicine, Istanbul Medeniyet University, Dumlupinar Mahallesi, Istanbul, Turkey; 15 National Medical Research Center of Cardiology, Moscow, Russia; 16 Faculty of Medicine, Universidad Peruana Cayetano Heredia, Lima, Peru; 17 Academic Section of Geriatric Medicine, Glasgow Royal Infirmary, University of Glasgow, Glasgow, United Kingdom; 18 Beijing Hypertension League Institute, Beijing, China; 19 Department of Medicine, Aga Khan University, Karachi, Pakistan

## Abstract

**Importance:**

Psychosocial stress is considered a modifiable risk factor for stroke. Given the prevalence of chronic and acute exposure to stress, it represents a potentially attractive target for population-health interventions.

**Objectives:**

To determine the association of psychosocial stress with the risk of acute stroke and explore factors that might modify the association of stress with risk of acute stroke in a large international population.

**Design, Setting, and Participants:**

INTERSTROKE is an international retrospective case-control study of risk factors for first acute stroke in 32 countries in Asia, North and South America, Europe, Australia, the Middle East, and Africa. A total of 13 462 patients with stroke and 13 488 matched controls were recruited between January 11, 2007, and August 8, 2015. The present analyses were performed from June 1 to 30, 2021, and included 13 350 cases and 13 462 controls with available data on psychosocial stress.

**Exposures:**

Psychosocial stress and occurrence of stressful life events within the preceding year were measured using a standardized questionnaire of self-reported stress at home and work.

**Main Outcomes and Measures:**

The association of stress with acute stroke and its subtypes was examined using multivariable conditional logistic regression and factors that might modify the association, particularly self-reported locus of control.

**Results:**

Among 26 812 participants included in the analysis, the mean (SD) age of cases was 62.2 (13.6) years; that of controls, 61.3 (13.3) years; 7960 cases (59.6%) and 8017 controls (59.6%) were men. Several periods of stress and permanent stress were reported for 2745 cases (20.5%) and 1933 controls (14.4%), with marked regional variation in prevalence, with the lowest in China (201 of 3981 [5.0%] among controls and 364 of 3980 [9.1%] among cases) and highest in South East Asia (233 of 855 [26.1%] among controls and 241 of 782 [30.8%] among cases). Increased stress at home (odds ratio [OR], 1.95 [95% CI, 1.77-2.15]) and at work (OR, 2.70 [95% CI, 2.25-3.23]) and recent stressful life events (OR, 1.31 [95% CI, 1.19-1.43]) were associated with an increased risk of acute stroke on multivariable analyses (vs no self-reported stress). Higher locus of control at home was associated with a reduced odds of all stroke (OR, 0.73 [95% CI, 0.68-0.79]), and higher locus of control both at work and at home were associated with a lower odds of acute stroke and significantly diminished the association with stress at work (OR, 2.20 [95% CI, 1.88-2.58]; *P* = .008 for interaction) and home (OR, 1.69 [95% CI, 1.44-1.98]; *P* < .001 for interaction) for acute stroke.

**Conclusions and Relevance:**

Psychosocial stress is a common risk factor for acute stroke. The findings of this case-control study suggest that higher locus of control is associated with lower risk of stroke and may be an important effect modifier of the risk associated with psychosocial stress.

## Key Points

**Questions:**

Is psychosocial stress associated with an increased risk of acute stroke, and does higher locus of control modify this risk?

**Findings:**

In this international case-control study of risk factors for first stroke in 26 812 participants, self-reported psychosocial stress was associated with increased risk of all stroke. Higher locus of control at work and home was associated with diminished magnitude of context-specific psychosocial stress and odds of acute stroke.

**Meaning:**

These findings suggest that higher locus of control may be an effect modifier of the risk for stroke associated with psychosocial stress.

## Teaser

This case-control study evaluates the associations of different psychosocial stressors with the risk of stroke in different populations and assesses whether factors such as locus of control are associated with modified risk.

## Introduction

Self-reported psychosocial stress has been reported to be an independent risk factor for stroke and myocardial infarction.^1,2,3,4^ Hence, some guidelines for cardiovascular disease prevention recommend screening for psychosocial stress in high-risk patients.^5,6^

Long-term exposure to increased stress has been associated with development of atherosclerosis and small-vessel disease (ie, intermediate phenotype), and short-term increases in stress have been reported as a trigger for acute cardiovascular events.^7,8,9,10^ The strength of the association of exposure to chronic stress with cardiovascular disease that has been reported in prospective cohort studies^11^ has generally been lower than the association of short-term increases in stress with triggering cardiovascular events that has been reported in case-control studies.^3^

Although reasonable consensus exists that psychosocial stress is a risk factor for stroke, no convincing interventions have been proven to reduce both stress and the risk of stroke. Consequently, there is debate about the logic of investing in public health interventions to target stress management and prevention.^12,13^ Although exposure to stress may have limited modifiability for many people and situations, there may be other opportunities to mitigate the association of stress and cardiovascular risk, such as enhancing coping strategies or environmental factors to mitigate the impact of stress (locus of control), which may be an important effect modifier.

The INTERSTROKE study offers an opportunity to evaluate the association of a recent exposure to psychosocial stress (ie, the past year) with stroke risk in an international population. The INTERSTROKE investigators^4^ have previously reported on the association between global psychosocial stress and stroke, reporting an increased risk of stroke associated with global psychosocial stress. The aim of the present analysis of the INTERSTROKE study was to evaluate the associations of different psychosocial stressors with the risk of stroke in different populations (characterized by age, sex, region, and self-reported ethnicity) and to consider whether factors such as locus of control are associated with modified risk.

## Methods

INTERSTROKE is an international case-control study of risk factors for first stroke. The methods have been described previously.^4^ Patients (13 462 with stroke and 13 488 matched controls) were recruited between January 11, 2007, and August 8, 2015. For the current analyses, we included 13 350 cases and 13 462 controls (13 350 matched pairs) with available data on psychosocial stress. Each case was matched for sex and age (±5 years) with a control from the same center or catchment area. Cases comprised patients who presented with a first ischemic or hemorrhagic acute stroke (confirmation by computed tomography or magnetic resonance imaging of the brain) and were enrolled within 72 hours of hospital admission or 5 days of symptom onset. Stroke severity was measured using the modified Rankin Scale at the time of recruitment and at 1-month follow-up. Control participants were either community based or hospital based. Hospital-based controls were patients admitted to a hospital or those attending an outpatient clinic for disorders or procedures not related to stroke or visitors or relatives of other inpatients as previously described (eMethods 1 in Supplement 1).^4^ The study was approved by the ethics committees at all participating centers. Written informed consent was obtained from participants or their proxies. This study adhered to the Strengthening the Reporting of Observational Studies in Epidemiology (STROBE) reporting guidelines.

### Measurement of Stress and Locus of Control

We measured psychosocial and psychological stress in the preceding year using a standardized questionnaire with questions relating to stress at home, work stress, financial stress, and stressful life events (eMethods 2 in Supplement 1). The questionnaire was translated from English to the local language, administered in the local language, and back translated after administration. Stress was defined as feeling irritable or filled with anxiety or as having sleeping difficulties as a result of conditions at work or at home. Cases were instructed to respond as they would before the stroke event. Participants were asked to grade how often they felt stress at work or home in the previous year. Home stress and work stress were graded as follows: never, some of the time, several periods of stress, and permanent stress. Because stress at work and at home were highly intercorrelated and because only 5266 cases and 5614 controls were currently working, we also generated a summary measure of general stress at home and/or in the workplace, similar to that of the INTERHEART study.^3^ This summary measure combined stress at work, home, or both and was categorized as follows: never, some periods at home or at work, several periods at home or at work, and permanent stress at home or at work. To assess whether there was an additive association regarding home stress and work stress among those who were working, a composite variable was derived. This composite variable of home and work stress was categorized as follows: neither work nor home stress, mild (some of the time) work or home stress, mild work and home stress, severe (several periods or permanent) home or work stress, and severe work and home stress. Financial stress was categorized into 3 levels: little or none, moderate, and high or severe. Work stress and home stress were categorized into 3 levels: never, some of the time, and several periods or permanent. Participants were also asked if they experienced specified stressful life events in the previous year.

Locus of control was assessed by responses to a 6-item scale measuring perceived control over what happens at work and in life, positive outlook for the future, perception of fairness, life changes during the past decade, and whether participants have given up on life improvements (eMethods 3 in Supplement 1). This scale has been used in the INTERHEART study^3^ and others.^14^ This 6-item scale measured self-reported locus of control in life and at work using single-item questions. To assess perceived life control, participants were asked to respond to the following statement, “I feel what happens in my life is often determined by factors beyond my control” on a 5-point Likert scale. To assess perceived work control, participants were asked to respond to the following statement, “At work, I feel I have control over what happens in most situations” on a 5-point Likert scale, with higher scores indicating strongly agree.

### Measurement of Other Risk Factor Exposures

Standardized questionnaires were used to collect data on baseline demographic characteristics, lifestyle stroke risk factors, and characteristics of acute stroke from all cases and controls. Ischemic stroke subtype was based on clinical assessment (baseline and 1 month), neuroimaging (baseline), and results of tests to determine stroke etiology. Physical measurements (eg, blood pressure) were recorded in a standardized manner. A modified Rankin Scale score was collected at 3 points for cases (preadmission, time of interview, and 1-month follow-up) and 1 point for controls (time of interview). Hypertension was defined as a composite of self-reported hypertension and a blood pressure reading of greater than 140/90 mm Hg at recruitment. We measured depressive symptoms by asking whether during the past 12 months the participant had felt sad, blue, or depressed for 2 or more consecutive weeks. Those who responded yes were asked to complete a 7-item questionnaire about associated symptoms (eMethods 3 in Supplement 1).

### Statistical Analysis

Data were analyzed from June 1 to 30, 2021. Simple associations were assessed with frequency tables and Pearson χ^2^ tests for 2 independent proportions. We used multivariable conditional logistic regression to evaluate the association between the following exposures with stroke: general stress, work stress, home stress, a composite of home and work stress, stressful life events (discrete and cumulative events), and financial stress. We used conditional logistic regression–matched case-control pairs for primary analysis of all stroke. All conditional analyses were stratified on the matching criteria (ie, controls were matched for sex and age [±5 years] with cases; age matching was extended [±10 years] for participants older than 90 years). We adjusted for covariates in 5 sequential models. Model 1 was adjusted for age (and matched for sex and center). Model 2 (the primary model) was additionally adjusted for occupation, educational level, and wealth index. Model 3 was additionally adjusted for diet, physical activity, alcohol consumption, and smoking, which was a model to explore variables potentially along the causal pathway mediating the association of stress and stroke. Model 4 was additionally adjusted for body mass index, waist-to-hip ratio, hypertension, atrial fibrillation, and diabetes. Model 5 was additionally adjusted for depressive symptoms. A sensitivity analysis was performed adjusting for the primary model (model 2) and other stress domains.

We completed an analysis to explore whether locus of control modified the association of stress and risk of stroke, where we derived a binary variable for locus of control, based on response to questions that signified high life control (strongly disagree or disagree), low life control (neutral response, agree, or strongly agree), high work control (strongly agree/agree) and low work control (neutral response, disagree, or strongly disagree) (eMethods 3 in Supplement 1). In these analyses (and other subgroup analysis), we used unconditional regression models, which additionally adjusted for sex and center (random effect) to our primary model. A likelihood ratio test was used to test for interaction between risk factor and subgroups (eg, regions). Two-sided *P* < .05 for interaction was considered statistically significant. Subgroup analyses were exploratory in nature, and we did not alter the threshold of the *P* value. Statistical analyses were performed using R, version 3.4.2 (R Project for Statistical Computing).

## Results

**Figure 1.**
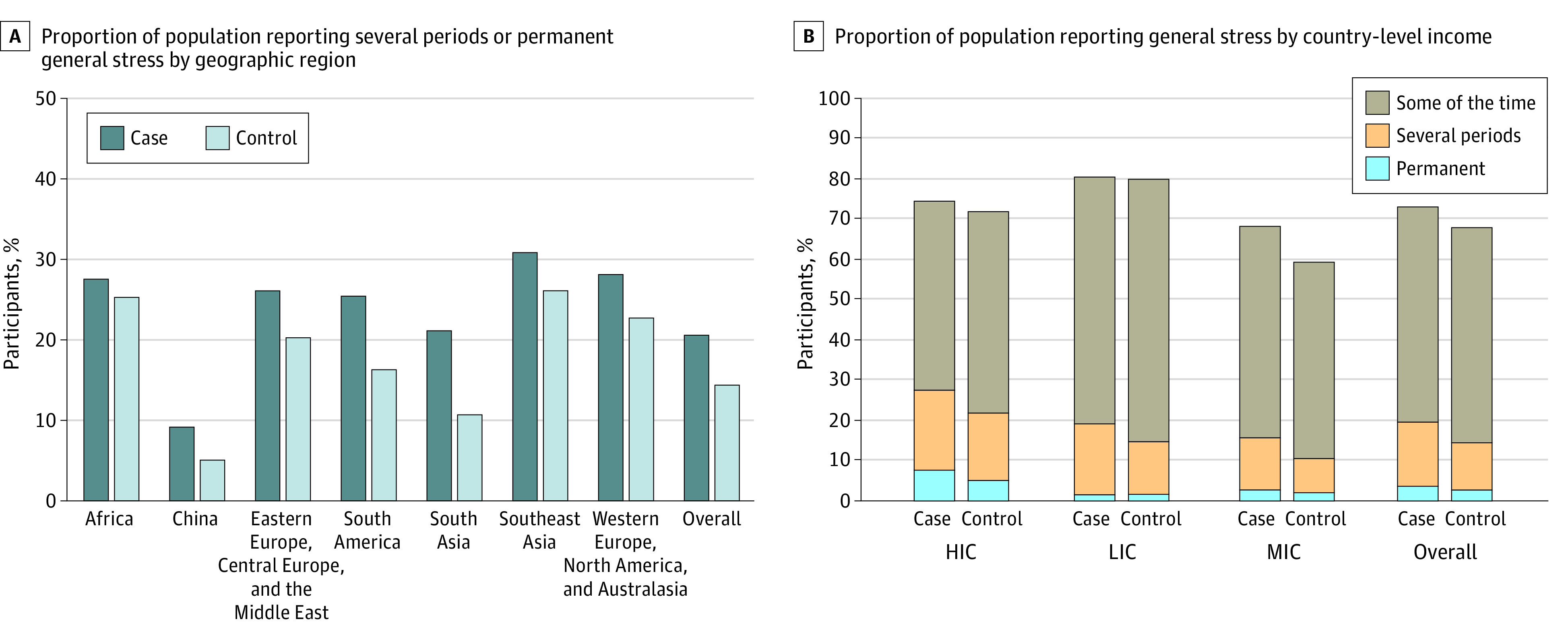
Prevalence of General Stress by Country Region and Income Level For regions, participants include 267 cases and 246 controls in Africa; 364 cases and 201 controls in China; 363 cases and 282 controls in Eastern and Central Europe and the Middle East; 370 cases and 241 controls in South America; 604 cases and 306 controls in South Asia; 241 cases and 223 controls in Southeast Asia; and 536 cases and 434 controls in Western Europe, North America, and Australasia. For country income level, participants include 2415 cases and 2333 controls in high-income countries (HIC); 2766 cases and 2750 controls in low-income countries (LIC); and 4242 cases and 3748 controls in middle-income countries (MIC).

A total of 26 812 participants were included, comprising 13 350 cases and 13 462 controls. The mean (SD) age of cases was 62.2 (13.6) years; that of controls, 61.3 (13.3) years. Among cases, 7960 (59.6%) were men and 5390 (40.4%) were women; among controls, 8017 (59.6%) were men and 5445 (40.4%) were women. The Table presents baseline characteristics among controls and cases by general stress level categories (ie, stress at home, work, or both). Individuals with high general stress were younger (mean [SD] age, 57.5 [13.7] vs 62.6 [13.2] years), had attained a higher number of years in formal education (postsecondary school, 1576 of 4678 [33.7%] vs 5279 of 22 134 [23.9%]), had higher body mass index (calculated as weight in kilograms divided by height in meters squared; mean [SD], 26.4 [5.5] vs 25.6 [4.8]), and were less physically active (656 of 2593 [25.3%] vs 1673 of 8199 [20.4%]). eTable 1 in Supplement 1 presents the association of cardiovascular risk factors with general stress. Figure 1 demonstrates the frequency of general stress levels by region and country income level. The lowest frequency of several periods of stress or permanent stress was reported in China (201 of 3981 [5.0%] among controls and 364 of 3980 [9.1%] among cases). The highest frequency of several periods of stress or permanent stress was reported in South East Asia (223 of 855 [26.1%] among controls and 241 of 782 [30.8%] among cases). High-income countries reported higher percentages of permanent stress (248 of 3242 cases [7.6%] and 165 of 3246 controls [5.1%]) compared with middle- or low-income countries (eg, 53 of 3437 cases [1.5%] and 57 of 3420 controls [1.7%] in low-income countries) (Figure 1).

**Table.** Cardiovascular Risk Factors, Educational Level, and Other Psychosocial Variables by General Stress Level^a^

Characteristic	Cases (n = 13 350)			Controls (n = 13 462)		
	Stress level		*P* value^b^	Stress level		*P* value^b^
	None or some periods (n = 10 605)	Several periods or permanent (n = 2745)		None or some periods (n = 11 529)	Several periods or permanent (n = 1933)	
Age, mean (SD), y	63.3 (13.3)	57.8 (13.6)	<.001	62.0 (13.1)	57.1 (13.8)	<.001
Sex						
Women	4312 (40.7)	1078 (39.3)	.19	4608 (40.0)	837 (43.3)	.006
Men	6293 (59.3)	1667 (60.7)		6921 (60.0)	1096 (56.7)	
Educational level						
None	1804 (17.0)	343 (12.5)	<.001	1422 (12.3)	209 (10.8)	<.001
1-8 y	4106 (38.7)	793 (28.9)		3835 (33.3)	451 (23.3)	
9-12 y	2704 (25.5)	796 (29.0)		2979 (25.8)	510 (26.4)	
Trade school, college, or university	1990 (18.8)	813 (29.6)		3289 (28.5)	763 (39.5)	
Occupation						
Professional	989 (9.3)	377 (13.7)	<.001	1855 (16.1)	408 (21.1)	<.001
Skilled or general laborer	3491 (32.9)	1010 (36.8)		3916 (34.0)	633 (32.7)	
Housewife or farmer	4172 (39.3)	665 (24.2)		3617 (31.4)	418 (21.6)	
Police, military, business, or clerical	1049 (9.9)	427 (15.6)		1303 (11.3)	291 (15.1)	
Disability or social security	299 (2.8)	51 (1.9)		259 (2.2)	43 (2.2)	
Other	602 (5.7)	214 (7.8)		568 (4.9)	139 (7.2)	
Physical activity at work, mainly sedentary^c^	706 (19.1)	384 (25.6)	<.001	967 (21.5)	272 (24.9)	<.001
History of alcohol consumption						
Never	6520 (61.5)	1349 (49.1)	<.001	7279 (63.1)	922 (47.7)	<.001
Former	1084 (10.2)	374 (13.6)		1241 (10.8)	284 (14.7)	
Low or moderate^d^	1620 (15.3)	608 (22.1)		1902 (16.5)	524 (27.1)	
High or binge^d^	1368 (12.9)	407 (14.8)		1097 (9.5)	202 (10.5)	
History of smoking or current smoker	3098 (29.2)	942 (34.3)	<.001	2597 (22.5)	418 (21.6)	.39
mAHEI Score, mean (SD)^e^	23 (6)	23 (7)	.001	23 (6)	24 (7)	<.001
Body mass index, mean (SD)^f^	25.6 (4.8)	26.6 (5.0)	<.001	25.5 (4.7)	26.2 (5.0)	<.001
Waist-to-hip ratio, mean (SD)	0.93 (0.08)	0.95 (0.09)	<.001	0.92 (0.08)	0.93 (0.08)	<.001
Hypertension^g^	7718 (72.8)	1980 (72.1)	.50	5430 (47.1)	938 (48.5)	.24
HDL cholesterol level, mean (SD), mg/dL^h^	42.9 (14.3)	42.9 (14.3)	.45	45.2 (15.1)	44.8 (15.4)	.02
Non-HDL cholesterol level, mg/dL^h^	137.5 (44.8)	141.7 (46.7)	<.001	140.5 (42.5)	139.8 (41.7)	.61
Diabetes^i^	2956 (27.9)	787 (28.7)	.39	2567 (22.3)	387 (20.0)	.03
History of atrial fibrillation or flutter	1054 (9.9)	261 (9.5)	.50	305 (2.6)	64 (3.3)	.10
Financial stress, moderate or severe	5059 (47.7)	1935 (70.5)	<.001	5234 (45.4)	1321 (68.3)	<.001
Home stress						
Never	4212 (39.7)	194 (7.1)	<.001	5041 (43.7)	172 (8.9)	<.001
Some of the time	6393 (60.3)	650 (23.7)		6488 (56.3)	467 (24.2)	
Several periods or permanent	0	1901 (69.3)		0	1292 (66.8)	
Work stress^c^						
Never	1273 (32.4)	64 (4.1)	<.001	1572 (33.9)	49 (4.4)	<.001
Some of the time	2655 (67.6)	229 (14.8)		3071 (66.1)	185 (16.5)	
Several periods or permanent	0	1257 (81.1)		0	887 (79.1)	
Stressful life events						
1	2061 (19.4)	811 (29.5)	<.001	2137 (18.5)	554 (28.7)	<.001
≥2	939 (8.9)	853 (31.1)		961 (8.3)	561 (29.0)	
Depressive symptoms, sad in last 2 wk	1439 (13.6)	1007 (36.7)	<.001	1241 (10.8)	660 (34.1)	<.001
High locus of control	8954 (85.2)	1840 (67.8)	<.001	10 078 (87.6)	1440 (74.7)	<.001

Abbreviations: HDL, high-density lipoprotein; mAHEI, modified Alternative Healthy Eating Index.

^a^ Unless otherwise indicated, data are expressed as No. (%) of participants. Owing to missing data, percentages may not total 100 for each category.

^b^ Calculated using the Wilcoxon rank-sum test and Pearson χ^2^ test.

^c^ Includes respondents who were employed.

^d^ Low or moderate intake defined as 0 to 30 U/mo; high or binge, greater than 30 U/mo.

^e^ Scores range from 0 to 70, with higher scores indicating a healthier diet.

^f^ Calculated as weight in kilograms divided by height in meters squared.

^g^ Defined as a history of hypertension or blood pressure of greater than 140/90 mm Hg on admission.

^h^ To convert to mmol/L, multiply by 0.0259.

^i^ Indicates history of diabetes or hemoglobin A_1c_ level of at least 6.5%.

**Figure 2.**
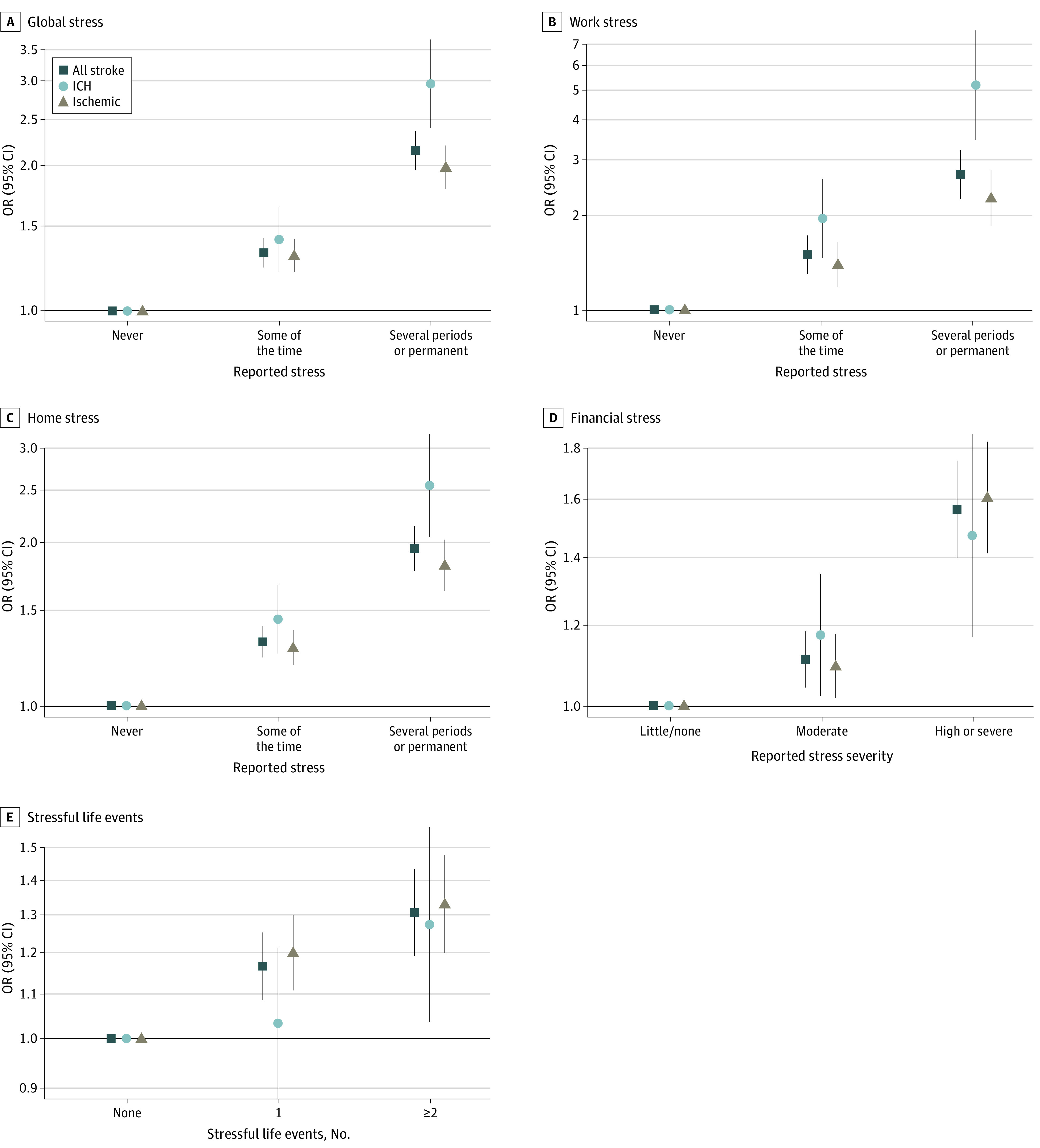
Association of Stress Types and Risk of Stroke Multivariable analysis adjusted for covariates in the primary model, including occupation, educational level, and wealth index. Work stress analysis included only those participants who were working. ICH indicates intracerebral hemorrhage; OR, odds ratio.

Overall, 1901 cases (14.2%) reported several periods of home stress or permanent home stress compared with 1292 controls (9.6%). Data were missing for 112 participants (0.4%). Having several periods of home stress or permanent home stress was associated with increased risk of all stroke (odds ratio [OR], 1.95 [95% CI, 1.77-2.15]), ischemic stroke (OR, 1.82 [95% CI, 1.63-2.03]), and intracerebral hemorrhage (OR, 2.55 [95% CI, 2.05-3.18]) on multivariable analysis (Figure 2 and eFigure and eTable 2 in Supplement 1). A total of 5266 cases (39.1%) and 5614 controls (41.6%) were employed. We had missing data for work stress for 175 participants (1.6%) who were working. During the previous year, 1214 cases who were working (23.1%) experienced several periods of work stress or permanent work stress compared with 865 controls (15.4%). Several periods of work stress or permanent work stress was associated with increased odds of all stroke (OR, 2.70 [95% CI, 2.25-3.23]), ischemic stroke (OR, 2.27 [95% CI, 1.85-2.78]), and intracerebral hemorrhage (OR, 5.20 [95% CI, 3.48-7.77]) on multivariable analysis (Figure 2 and eFigure and eTable 2 in Supplement 1). There was a higher magnitude of association of several periods of home stress or permanent home stress among those who experienced several periods of work stress or permanent work stress (OR, 3.64 [95% CI, 2.79-4.75]) (eTable 2 in Supplement 1).

Any stressful life event was also associated with increased odds of stroke (OR, 1.17 [95% CI, 1.09-1.25]), with higher magnitude of association for 2 or more stressful life events (OR, 1.31 [95% CI, 1.19-1.43]) (Figure 2 and eTable 2 in Supplement 1). Of the stressful life events, major intrafamily conflict (OR, 1.77 [95% CI, 1.60-1.96]), marital separation or divorce (OR, 1.33 [95% CI, 1.07-1.66]), death of a spouse (OR, 1.35 [95% CI, 1.11-1.63]), and violence (OR, 1.26 [95% CI, 1.03-1.54]) were individually associated with a significantly increased odds of all stroke (eTable 3 in Supplement 1).

**Figure 3.**
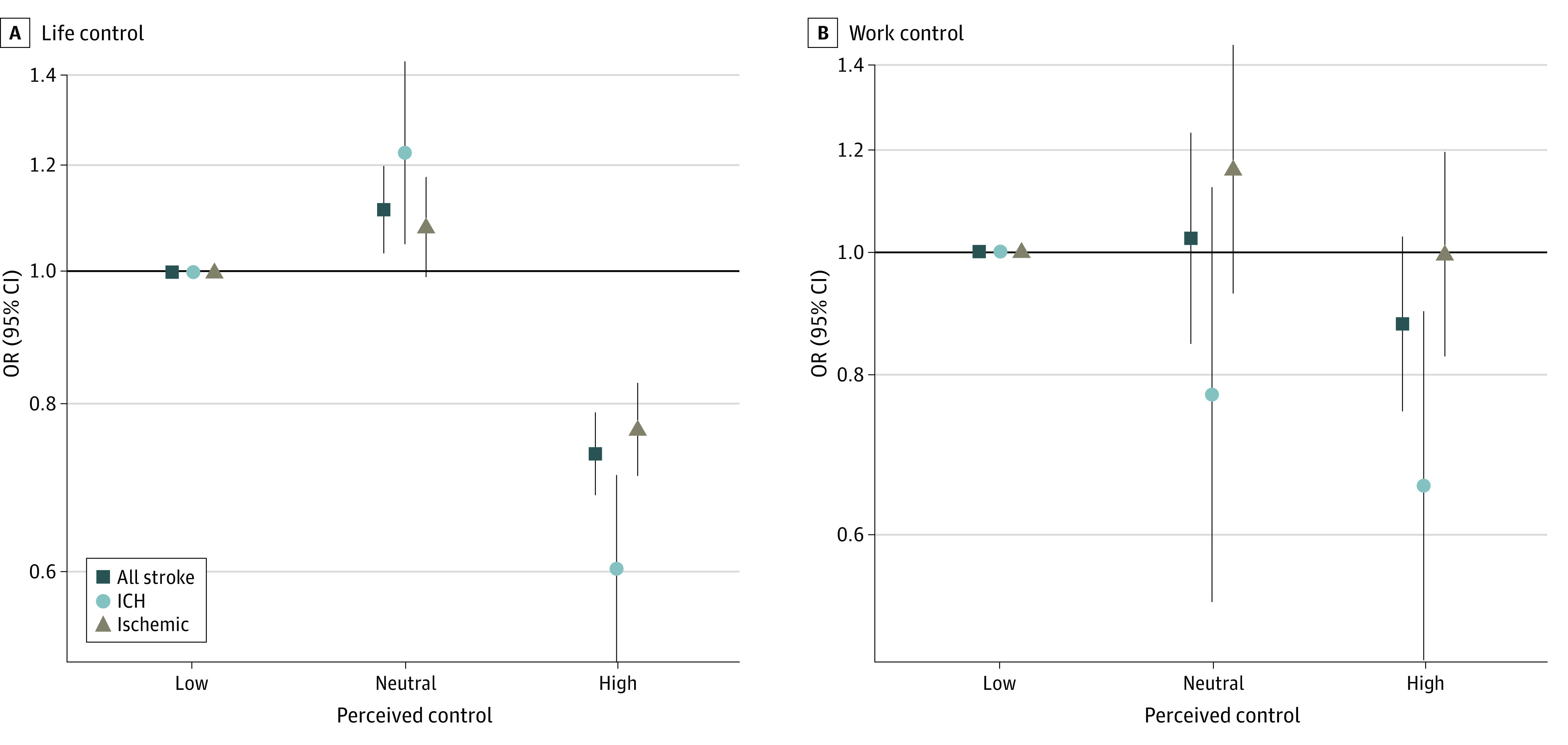
Association of Life and Work Control and Risk of Stroke Multivariable analysis adjusted for covariates in the primary model, including occupation, educational level, and wealth index. Work control analysis included only those participants who were working. ICH indicates intracerebral hemorrhage; OR, odds ratio.

Higher locus of control at home was associated with a reduced odds of all stroke (OR, 0.73 [95% CI, 0.68-0.79]). However, higher locus of control at work was not associated with risk of stroke (OR, 0.90 [95% CI, 0.80-1.01]) (Figure 3). In those who experienced several periods of home stress or permanent home stress, high life control was associated with lower odds for all stroke (OR, 1.69 [95% CI, 1.44-1.98]) compared with low life control (OR, 2.40 [95% CI, 2.11-2.72]) (*P* < .001 for interaction) (eTable 4 in Supplement 1). Similarly, among those who experienced several periods of work stress or permanent work stress, high work control was associated with lower odds for all stroke (OR, 2.20 [95% CI, 1.88-2.58]) compared with low work control (OR, 2.70 [95% CI, 2.16-3.37]) (*P* = .008 for interaction) (eTable 4 in Supplement 1). The interaction between work control and home stress was not significant (*P* = .02 for interaction). High life control also modified the association of death or major illness of a family member and risk of stroke (OR for high control, 0.93 [95% CI, 0.82-1.05]; OR for low control, 0.99 [95% CI, 0.91-1.08]; *P* = .007 for interaction), but not other discrete life events. eTable 5 in Supplement 1 reports an analysis stratified by control.

eTable 6 in Supplement 1 presents an analysis stratified by region, demonstrating a significant association between several periods of general stress or permanent general stress with all stroke in all regions other than Africa (*P* < .001 for interaction). The association between several periods of global stress or permanent global stress and all stroke was not significant within Africa for ischemic stroke (OR, 0.86 [95% CI, 0.57-1.31]) but was associated with a significant increase in intracerebral hemorrhage (OR, 2.00 [95% CI, 1.04-3.84]) (eTable 6 in Supplement 1).

eTable 7 in Supplement 1 presents analysis stratified by sex, demonstrating a consistent association between chronic stress domains and all stroke. The association between some stressful life events and all stroke varied by sex; major intrafamily conflict was associated a higher risk of stroke in men (OR, 1.96 [95% CI, 1.72-2.23]) compared with women (OR, 1.64 [95% CI, 1.43-1.88]) (*P* = .02 for interaction), and other stressful life events were associated with higher odds of all stroke in men (OR, 1.69 [95% CI, 1.44-1.98]) compared with women (OR, 1.27 [95% CI, 1.07-1.51]) (*P* = .008 for interaction).

eTable 8 in Supplement 1 presents an analysis stratified by age group (<45, 45-65, and >65 years). The association of home stress and work stress with all stroke was consistent across age groups. Financial stress was associated with the highest odds of all stroke in those aged 45 to 65 years (OR, 1.85 [95% CI, 1.58-2.17]) (*P* < .001 for interaction). The association between other stress and all stroke also varied by age group, with highest odds of all stroke in those younger than 45 years (OR, 2.25 [95% CI, 1.59-3.17]). Business failure or loss of crop was associated with highest odds of all stroke in those younger than 45 years (OR, 1.13 [95% CI, 0.79-1.61]) (*P* = .05 for interaction). The association of discrete stressful life events (with the exception of business failure or loss of crop) and all stroke was consistent across age groups.

eTable 9 in Supplement 1 presents a sensitivity analysis reporting the association of stress domains and stroke adjusted for the primary model and the other stress domains. There was no material difference in conclusions.

## Discussion

Our study found that self-reported psychosocial stress within the previous 12 months was associated with increased risk of all stroke, ischemic stroke, and hemorrhagic stroke. This association was consistent for all stress domains, including work stress, home stress, and financial stress. The association was independent of socioeconomic status, occupation, and educational level and remained significant after adjustment for cardiovascular risk factors, suggesting that some of the association may be independent of other cardiovascular risk factors (eg, blood pressure, smoking, unhealthy diet). We report that higher locus of control at work and home diminished the magnitude of association between context-specific psychosocial stress and odds of acute stroke.

Our findings from the INTERSTROKE study add to prior evidence, which has come predominantly from studies in high-income countries.^1,2^ The association between stress and stroke was first reported in the early 1990s, with observational studies identifying that stroke had a higher incidence in men with high self-perceived stress ratings.^15^ Since then, numerous studies have evaluated the association of stress and stroke, and meta-analyses of these studies^1^ report a significant increase in stroke risk and other cardiovascular events. Our estimate of increased risk associated with global stress is consistent with a systematic review of prospective cohort studies^16^ and case-controls studies,^3^ as is the finding that stress is a stronger risk factor for intracerebral hemorrhage than for ischemic stroke. In general, case-control studies, other than our present analysis, have reported higher magnitudes of risk than prospective cohort studies, which may relate to recall bias in case-control studies with spurious inflation of ORs. However, it may also relate to differences in the type of association, in that prospective cohort studies often measure stress at an interval that is remote from the cardiovascular event, whereas case-controls studies capture recent exposure to stress, which may reflect both chronic exposure and acute triggering increases in stress.^17^ Therefore, a higher magnitude of risk, if the association of stress and stroke is truly causal, might be expected in case-control studies, which is unrelated to biases.

The European Society of Cardiology^5^ and American Heart Association^6^ guidelines for cardiovascular prevention include stress as a potentially modifiable risk factor for stroke and coronary heart disease. The European Society of Cardiology makes a class IA recommendation to integrate stress management and counselling on psychosocial risk factors for individuals at very high cardiovascular risk.^5^ However, optimal approaches to managing and preventing psychosocial stress are uncertain. A Cochrane review of psychological interventions for coronary heart disease^18^ reported a significant reduction in cardiovascular mortality in intervention groups compared with control groups but noted the heterogeneity of interventions and low quality of evidence. Interventions included in studies were multicomponent, and the most common domains were cognitive behavioral therapy, relaxation techniques, client-led discussion, stress management, exercise regimens, and anger management.^18^ The World Health Organization^19^ also released guidance on management of conditions specifically related to stress and a mental health intervention guide in 2013. However, stress interventions face numerous challenges, including lack of availability of resources, particularly in lower income countries (eg, access to cognitive behavioral therapy), and ongoing stressors that will not be modifiable for the individual (eg, chronic poverty, being a refugee, or ongoing armed conflict).^13,20^ Our findings would support the need to develop generalizable, effective, and feasible interventions to reduce psychosocial stressors, whereas relieving conditions related to poverty and conflicts will rest with political decisions.

Several studies have shown that low control in the work place is a risk factor for cardiovascular disease.^21,22,23^ There is also evidence that job strain (high demands and low control) is associated with increased risk of ischemic stroke.^24^ We report a significant interaction between locus of control and stress at home and work, which is consistent with findings of the INTERHEART study.^3^ We observed a source-specific interaction, with locus of control at work a stronger effect modifier of the association with work stress (*P* = .008 for interaction) and stroke (*P* = .02 for interaction) compared with home stress. These findings suggest that improvement in locus of control might be an important target for mitigating adverse cardiovascular outcomes associated with psychosocial stress. In particular, workplace intervention may be promising, based on the demand-control-support model of workplace and health association. This model hypothesizes that job demands may negatively impact employees’ health; however, employees may decrease stress by increasing control or developing supportive relationships within the workplace.^25^ Karasek et al^25^ proposed that interventions to reduce work strain be considered macro or organizational-level interventions or micro or individual-level interventions. A systematic review of organizational-level interventions^26^ suggests that participation interventions (eg, flexible working hours or problem-solving committees) at an organizational level may improve health outcomes. A companion systematic review of individual-level interventions^27^ demonstrated that task restructuring that increases demand or decreases control adversely affected employees’ health, supporting interventions to improve job control.

Several mechanisms have been proposed to explain the association between stress and stroke or cardiovascular risk. Acute stress may trigger an event by leading to activation of the sympathetic nervous system causing vasoconstriction and plaque rupture in vulnerable individuals.^7^ Chronic stress is hypothesized to cause dysregulation of the sympathetic system, endothelial dysfunction, and atherosclerosis.^28^ Stress may indirectly increase stroke risk by fostering unhealthy behaviors.^29^ We note that those who reported several periods of general stress or permanent general stress were more likely to be mainly sedentary, had higher body mass index, and more frequently reported high levels of alcohol intake. However, the association between general stress and stroke does not appear to be attenuated by the addition of behavioral risk factors in our findings (model 3, eFigure in Supplement 1).

### Limitations

This study has some limitations. Stress was self-reported, and the perception of stress may vary between individuals and may be subject to recall bias. Stress and depressive symptoms are common after stroke.^30,31^ To mitigate the potential for reverse-causation bias, cases were instructed to answer as they would before their stroke. In addition, the interpretation of questions on stress may vary by country and culture, making interpretation of regional variations in prevalence uncertain.^32^ However, this source of potential bias should not influence the magnitude of association, which is expected to be represented in cases and controls similarly.

## Conclusions

The findings of this international case-control study suggest association between psychosocial stress and stressful life events with increased risk of all stroke. This association is consistent for ischemic and hemorrhagic stroke types. Our findings suggest that the association between stress and stroke is consistent across age groups and geographic regions, with the possible exception of Africa. Locus of control and its relevant components, life control and work control, appear to be important effect modifiers. The association between locus of control and stroke appears to be contextual (eg, work control appears to modify the effect size of work stress on stroke risk). Locus of control warrants further evaluation as a potential target for public health interventions.

## Notes

Supplement 1. **eMethods 1.** Selection of Controls **eMethods 2.** Stress or Stressful Life Events Questionnaire **eMethods 3.** Locus of Control and Depression Questions **eTable 1.** Association of Cardiovascular Risk Factors With Odds of Several Periods or Permanent General Stress **eTable 2.** Prevalence of Stress Domains and Conditional Logistic Regression **eTable 3.** Association Between Stressful Life Events and Stroke Types (Conditional Logistic Regression) **eTable 4.** Composite of Stress and Control Prevalence and Unconditional Logistic Regression **eTable 5.** Association of Work and Home Stress With All Stroke Stratified by Control **eTable 6.** Several Periods of General Stress or Permanent General Stress Prevalence and Conditional Logistic Regression by Region **eTable 7.** Psychosocial Stress Prevalence and Conditional Logistic Regression by Sex **eTable 8.** Psychosocial Stress Prevalence and Conditional Logistic Regression by Age **eTable 9.** Association of Stress Domains and Stroke (Sensitivity Analysis) **eFigure.** Multivariate Logistic Regression Models (Conditional) **eAppendix.** INTERSTROKE Study Site Staff

Supplement 2.
Nonauthor Collaborators

Supplement 3.
Data Sharing Statement

## References

[1] Booth J, Connelly L, Lawrence M, . Evidence of perceived psychosocial stress as a risk factor for stroke in adults: a meta-analysis. BMC Neurol. 2015;15(1):233. doi:10.1186/s12883-015-0456-4 26563170 PMC4643520

[2] Lightbody CE, Clegg A, Patel K, . Systematic review and meta-analysis of psychosocial risk factors for stroke. Semin Neurol. 2017;37(3):294-306. doi:10.1055/s-0037-1603758 28759911

[3] Rosengren A, Hawken S, Ôunpuu S, ; INTERHEART Investigators. Association of psychosocial risk factors with risk of acute myocardial infarction in 11 119 cases and 13 648 controls from 52 countries (the INTERHEART study): case-control study. Lancet. 2004;364(9438):953-962. doi:10.1016/S0140-6736(04)17019-0 15364186

[4] O’Donnell MJ, Chin SL, Rangarajan S, ; INTERSTROKE Investigators. Global and regional effects of potentially modifiable risk factors associated with acute stroke in 32 countries (INTERSTROKE): a case-control study. Lancet. 2016;388(10046):761-775. doi:10.1016/S0140-6736(16)30506-2 27431356

[5] Piepoli MF, Hoes AW, Agewall S, ; ESC Scientific Document Group. 2016 European Guidelines on cardiovascular disease prevention in clinical practice: the Sixth Joint Task Force of the European Society of Cardiology and Other Societies on Cardiovascular Disease Prevention in Clinical Practice (constituted by representatives of 10 societies and by invited experts) developed with the special contribution of the European Association for Cardiovascular Prevention & Rehabilitation (EACPR). Eur Heart J. 2016;37(29):2315-2381. doi:10.1093/eurheartj/ehw106 27222591 PMC4986030

[6] Arnett DK, Blumenthal RS, Albert MA, . 2019 ACC/AHA Guideline on the Primary Prevention of Cardiovascular Disease: A Report of the American College of Cardiology/American Heart Association Task Force on Clinical Practice Guidelines. Circulation. 2019;140(11):e596-e646. doi:10.1161/CIR.0000000000000678 30879355 PMC7734661

[7] Mittleman MA, Mostofsky E. Physical, psychological and chemical triggers of acute cardiovascular events: preventive strategies. Circulation. 2011;124(3):346-354. doi:10.1161/CIRCULATIONAHA.110.968776 21768552 PMC3139921

[8] Padgett DA, Glaser R. How stress influences the immune response. Trends Immunol. 2003;24(8):444-448. doi:10.1016/S1471-4906(03)00173-X 12909458

[9] Smyth A, O’Donnell M, Lamelas P, Teo K, Rangarajan S, Yusuf S; INTERHEART Investigators. Physical activity and anger or emotional upset as triggers of acute myocardial infarction: the INTERHEART study. Circulation. 2016;134(15):1059-1067. doi:10.1161/CIRCULATIONAHA.116.023142 27753614

[10] Yao BC, Meng LB, Hao ML, Zhang YM, Gong T, Guo ZG. Chronic stress: a critical risk factor for atherosclerosis. J Int Med Res. 2019;47(4):1429-1440. doi:10.1177/0300060519826820 30799666 PMC6460614

[11] Richardson S, Shaffer JA, Falzon L, Krupka D, Davidson KW, Edmondson D. Meta-analysis of perceived stress and its association with incident coronary heart disease. Am J Cardiol. 2012;110(12):1711-1716. doi:10.1016/j.amjcard.2012.08.004 22975465 PMC3511594

[12] Macleod J, Davey Smith G. Psychosocial factors and public health: a suitable case for treatment? J Epidemiol Community Health. 2003;57(8):565-570. doi:10.1136/jech.57.8.565 12883057 PMC1732553

[13] Tol WA, Barbui C, Bisson J, . World Health Organization guidelines for management of acute stress, PTSD, and bereavement: key challenges on the road ahead. PLoS Med. 2014;11(12):e1001769. doi:10.1371/journal.pmed.1001769 25514024 PMC4267806

[14] Bobak M, Pikhart H, Rose R, Hertzman C, Marmot M. Socioeconomic factors, material inequalities, and perceived control in self-rated health: cross-sectional data from seven post-communist countries. Soc Sci Med. 2000;51(9):1343-1350. doi:10.1016/S0277-9536(00)00096-4 11037221

[15] Harmsen P, Rosengren A, Tsipogianni A, Wilhelmsen L. Risk factors for stroke in middle-aged men in Göteborg, Sweden. Stroke. 1990;21(2):223-229. doi:10.1161/01.STR.21.2.223 2305396

[16] Hemingway H, Marmot M. Evidence based cardiology: psychosocial factors in the aetiology and prognosis of coronary heart disease: systematic review of prospective cohort studies. BMJ. 1999;318(7196):1460-1467. doi:10.1136/bmj.318.7196.1460 10346775 PMC1115843

[17] Akpalu A, Gebregziabher M, Ovbiagele B, . Differential impact of risk factors on stroke occurrence among men versus women in West Africa. Stroke. 2019;50(4):820-827. doi:10.1161/STROKEAHA.118.022786 30879432 PMC6433514

[18] Whalley B, Rees K, Davies P, . Psychological interventions for coronary heart disease. Cochrane Database Syst Rev. 2011;(8):CD002902. doi:10.1002/14651858.CD002902.pub3 21833943

[19] World Health Organization. Guidelines for the Management of Conditions Specifically Related to Stress. WHO; 2013. 24049868

[20] Tol WA, Barbui C, van Ommeren M. Management of acute stress, PTSD, and bereavement: WHO recommendations. JAMA. 2013;310(5):477-478. doi:10.1001/jama.2013.166723 23925613

[21] Bosma H, Marmot MG, Hemingway H, Nicholson AC, Brunner E, Stansfeld SA. Low job control and risk of coronary heart disease in Whitehall II (prospective cohort) study. BMJ. 1997;314(7080):558-565. doi:10.1136/bmj.314.7080.558 9055714 PMC2126031

[22] Johnson JV, Stewart W, Hall EM, Fredlund P, Theorell T. Long-term psychosocial work environment and cardiovascular mortality among Swedish men. Am J Public Health. 1996;86(3):324-331. doi:10.2105/AJPH.86.3.324 8604756 PMC1380510

[23] Kivimäki M, Jokela M, Nyberg ST, ; IPD-Work Consortium. Long working hours and risk of coronary heart disease and stroke: a systematic review and meta-analysis of published and unpublished data for 603,838 individuals. Lancet. 2015;386(10005):1739-1746. doi:10.1016/S0140-6736(15)60295-1 26298822

[24] Fransson EI, Nyberg ST, Heikkilä K, . Job strain and the risk of stroke: an individual-participant data meta-analysis. Stroke. 2015;46(2):557-559. doi:10.1161/STROKEAHA.114.008019 25563644

[25] Karasek R, Baker D, Marxer F, Ahlbom A, Theorell T. Job decision latitude, job demands, and cardiovascular disease: a prospective study of Swedish men. Am J Public Health. 1981;71(7):694-705. doi:10.2105/AJPH.71.7.694 7246835 PMC1619770

[26] Egan M, Bambra C, Thomas S, Petticrew M, Whitehead M, Thomson H. The psychosocial and health effects of workplace reorganization, 1: a systematic review of organisational-level interventions that aim to increase employee control. J Epidemiol Community Health. 2007;61(11):945-954. doi:10.1136/jech.2006.054965 17933951 PMC2465601

[27] Bambra C, Egan M, Thomas S, Petticrew M, Whitehead M. The psychosocial and health effects of workplace reorganization, 2: a systematic review of task restructuring interventions. J Epidemiol Community Health. 2007;61(12):1028-1037. doi:10.1136/jech.2006.054999 18000123 PMC2465678

[28] Kershaw KN, Lane-Cordova AD, Carnethon MR, Tindle HA, Liu K. Chronic stress and endothelial dysfunction: the Multi-Ethnic Study of Atherosclerosis (MESA). Am J Hypertens. 2017;30(1):75-80. doi:10.1093/ajh/hpw103 27585566 PMC5155567

[29] Kouvonen A, Kivimäki M, Virtanen M, Pentti J, Vahtera J. Work stress, smoking status, and smoking intensity: an observational study of 46,190 employees. J Epidemiol Community Health. 2005;59(1):63-69. doi:10.1136/jech.2004.019752 15598729 PMC1763376

[30] Stein LA, Goldmann E, Zamzam A, . Association between anxiety, depression, and post-traumatic stress disorder and outcomes after ischemic stroke. Front Neurol. 2018;9:890. doi:10.3389/fneur.2018.00890 30450075 PMC6224432

[31] Bruggimann L, Annoni JM, Staub F, von Steinbüchel N, Van der Linden M, Bogousslavsky J. Chronic posttraumatic stress symptoms after nonsevere stroke. Neurology. 2006;66(4):513-516. doi:10.1212/01.wnl.0000194210.98757.49 16505303

[32] Steel Z, Silove D, Giao NM, . International and indigenous diagnoses of mental disorder among Vietnamese living in Vietnam and Australia. Br J Psychiatry. 2009;194(4):326-333. doi:10.1192/bjp.bp.108.050906 19336784

