# Supplementary material for: Association of Psychosocial Stress With Risk of Acute Stroke
Source: JAMA Netw Open. 2022 Dec 9;5(12):e2244836. doi: 10.1001/jamanetworkopen.2022.44836 (PMC9856236; doi:10.1001/jamanetworkopen.2022.44836)
Supplement: Supplement 1. — eMethods 1. Selection of Controls eMethods 2. Stress or Stressful Life Events Questionnaire eMethods 3. Locus of Control and Depression Questions eTable 1. Association of Cardiovascular Risk Factors With Odds of Several Periods or Permanent General Stress eTable 2. Prevalence of Stress Domains and Conditional Logistic Regression eTable 3. Association Between Stressful Life Events and Stroke Types (Conditional Logistic Regression) eTable 4. Composite of Stress and Control Prevalence and Unconditional Logistic Regression eTable 5. Association of Work and Home Stress With All Stroke Stratified by Control eTable 6. Several Periods of General Stress or Permanent General Stress Prevalence and Conditional Logistic Regression by Region eTable 7. Psychosocial Stress Prevalence and Conditional Logistic Regression by Sex eTable 8. Psychosocial Stress Prevalence and Conditional Logistic Regression by Age eTable 9. Association of Stress Domains and Stroke (Sensitivity Analysis) eFigure. Multivariate Logistic Regression Models (Conditional) eAppendix. INTERSTROKE Study Site Staff [file jamanetwopen-e2244836-s001.pdf]

Supplementary Online Content

Reddin C, Murphy R, Hankey GJ, et al; INTERSTROKE investigators. Association of psychosocial stress with risk of acute stroke. *JAMA Netw Open*. 2022;5(12):e2244836. doi:10.1001/jamanetworkopen.2022.44836

eMethods 1. Selection of Controls
eMethods 2. Stress or Stressful Life Events Questionnaire
eMethods 3. Locus of Control and Depression Questions
eTable 1. Association of Cardiovascular Risk Factors With Odds of Several Periods or Permanent General Stress
eTable 2. Prevalence of Stress Domains and Conditional Logistic Regression
eTable 3. Association Between Stressful Life Events and Stroke Types (Conditional Logistic Regression)
eTable 4. Composite of Stress and Control Prevalence and Unconditional Logistic Regression
eTable 5. Association of Work and Home Stress With All Stroke Stratified by Control
eTable 6. Several Periods of General Stress or Permanent General Stress Prevalence and Conditional Logistic Regression by Region
eTable 7. Psychosocial Stress Prevalence and Conditional Logistic Regression by Sex
eTable 8. Psychosocial Stress Prevalence and Conditional Logistic Regression by Age
eTable 9. Association of Stress Domains and Stroke (Sensitivity Analysis)
eFigure. Multivariate Logistic Regression Models (Conditional)
eAppendix. INTERSTROKE Study Site Staff

This supplementary material has been provided by the authors to give readers additional information about their work.

© 2022 Reddin C et al. *JAMA Network Open*.

eMethods 1. Selection of Controls

Controls:

1. Community-based control.
2. Relative of a patient from a non-cardiac ward.
3. Unrelated (not first degree relative) visitor of any patient.
4. Patients attending the hospital or outpatient clinic**:**

Preferred controls from hospital settings:

Patients attending the hospital or outpatients clinic for the following reasons:

1. Refraction and cataracts (excluding those presenting with acute visual loss).
2. Physical check-up.
3. Routine pap smear.
4. Routine breast exam.
5. Elective minor surgery for conditions that are not obviously related to stroke or its risk factors.
6. Elective orthopaedic surgery.

Acceptable controls from hospital settings:

Patients attending the hospital or outpatients clinic for the following reasons:

1. Outpatient fractures.
2. Arthritic complaints.
3. Plastic surgery.
4. Haemorrhoids, hernias, hydroceles.
5. Routine colon cancer screening.
6. Endoscopy.
7. Minor dermatological disorders.

© 2022 Reddin C et al. *JAMA Network Open*.

eMethods 2. Stress or Stressful Life Events Questionnaire

35. Have you experienced any of the following in the past year?

Marital separation/divorce	☐ No	☐ Yes	Major personal injury or illness	☐ No	☐ Yes
Loss of job/retirement	☐ No	☐ Yes	Death/major illness of a close family member	☐ No	☐ Yes
Loss of crop/ business failure	☐ No	☐ Yes	Death of a spouse	☐ No	☐ Yes
Violence	☐ No	☐ Yes	Other major stress____________ (if yes, please specify)	☐ No	☐ Yes
Major intra-family conflict	☐ No	☐ Yes			

36. For the following question, stress is defined as feeling irritable or filled with anxiety, or as having difficulties as a result of conditions at work or at home.

	Never Experienced Stress	Some Period of Stress	Several Periods of Stress	Permanent Stress
a) How often have you felt stress at work in the past year? (Mark here if not applicable: i.e. no longer working ☐)	☐	☐	☐	☐
b) How often have you felt stress at home in the past year?	☐	☐	☐	☐

c) What level of financial stress do you feel? ☐ Little/none ☐ Moderate ☐ High/Severe

d) How much autonomy do you have in organizing the events of your work day?

☐ None ☐ Little ☐ Moderate ☐ Substantial ☐ Complete ☐ Not Applicable

© 2022 Reddin C et al. *JAMA Network Open*.

eMethods 3. Locus of Control and Depression Questions

37. To what extent do you agree or disagree with the following statements:

	Strongly disagree	Disagree	Neutral	Agree	Strongly agree
a) At work, I feel I have control over what happens in most situations.	☐	☐	☐	☐	☐
b) I feel what happens in my life is often determined by factors beyond my control.	☐	☐	☐	☐	☐
c) Over the next 5-10 years, I expect to have more positive than negative experiences.	☐	☐	☐	☐	☐
d) I often have the feeling I am being treated unfairly.	☐	☐	☐	☐	☐
e) In the past 10 years my life has been full of changes without my knowing what will happen next.	☐	☐	☐	☐	☐
f) I gave up trying to make big improvements in my life a long time ago.	☐	☐	☐	☐	☐

38. During the past twelve months, was there ever a time when you felt sad, blue, or depressed for two weeks or more in a row?

☐ No ☐ Yes → **During those times, did you:**

	No	Yes
a) Lose interest in most things like hobbies, work or activities that usually give you pleasure?	☐	☐
b) Feel tired or low on energy?	☐	☐
c) Gain or lose weight?	☐	☐
d) Have more trouble falling asleep than you usually do?	☐	☐
e) Have more trouble concentrating than usual?	☐	☐
f) Think a lot about death (either your own, someone else's, or death in general)	☐	☐
g) Feel down on yourself, no good or worthless?	☐	☐

© 2022 Reddin C et al. *JAMA Network Open*.

eTable 1. Association of Cardiovascular Risk Factors With Odds of Several Periods or Permanent General Stress

		**All**	**Ischaemic**	**ICH**
**Predictor Variable**		**Odds Ratio (95% CI)**	**Odds Ratio (95% CI)**	**Odds Ratio (95% CI)**
Hypertension				
	No	1.0	1.0	1.0
		1.26 (1.18-1.35)	1.26 (1.17-1.37)	1.27 (1.1-1.46)
Systolic BP (admission)				
	1 mmHg change	1 (1-1.01)	1 (1-1)	1.01 (1.01-1.01)
Diastolic BP (admission)				
	1 mmHg change	1.01 (1-1.01)	1 (1-1.01)	1.01 (1.01-1.02)
AHEISCORE				
	aheiscore	1.01 (1.01-1.02)	1.01 (1.01-1.02)	1.01 (1-1.02)
BMI				
	BMI	1.03 (1.02-1.03)	1.03 (1.02-1.04)	1.01 (0.99-1.02)
Diabetes				
	No	1.0	1.0	1.0
	Yes	1.21 (1.11-1.32)	1.22 (1.11-1.35)	1.01 (0.79-1.27)

© 2022 Reddin C et al. *JAMA Network Open*.

eTable 2. Prevalence of Stress Domains and Conditional Logistic Regression

Risk Factors	Controls: N	Ischemic: N	ICH: N	Odds Ratio (95% CI)		
				**Multivariate analysis[1]**		
	**Prevalence**			**All stroke**	**Ischaemic stroke**	**ICH**
**General Stress**						
Never	4,217	2,772	760	1.0	1.0	1.0
Some of the time	7,345	5,425	1,615	1.32 (1.23-1.42)	1.3 (1.2-1.41)	1.41 (1.2-1.65)
Several Periods/Permanent	1,944	2,101	633	2.16 (1.97-2.37)	1.99 (1.79-2.21)	2.97 (2.4-3.67)
**Work Stress[2]**						
Never	1,548	885	308	1.0	1.0	1.0
Some of the time	3,165	1,953	767	1.5 (1.3-1.72)	1.39 (1.18-1.64)	1.95 (1.47-2.61)
Several Periods/Permanent	871	895	313	2.7 (2.25-3.23)	2.27 (1.85-2.78)	5.2 (3.48-7.77)
**Home Stress**						
Never	5,237	3,447	963	1.0	1.0	1.0
Some of the time	6,981	5,392	1,625	1.31 (1.23-1.4)	1.28 (1.19-1.38)	1.44 (1.25-1.67)
Several Periods/Permanent	1,301	1,469	423	1.95 (1.77-2.15)	1.82 (1.63-2.03)	2.55 (2.05-3.18)
**Composite of Home and Work Stress**						
Neither	1,247	674	233	1.0	1.0	1.0
Mild work or home stress	1,063	656	228	1.51 (1.27-1.8)	1.4 (1.15-1.72)	1.93 (1.36-2.73)
Both mild	2,181	1,315	540	1.67 (1.41-1.98)	1.5 (1.23-1.83)	2.46 (1.72-3.5)
One severe	850	800	280	2.72 (2.23-3.32)	2.24 (1.78-2.81)	5.51 (3.57-8.52)
Both severe	243	288	107	3.64 (2.79-4.75)	2.95 (2.18-3.99)	7.68 (4.25-13.87)
**Financial Stress**						
Little/none	6,943	5,144	1,202	1.0	1.0	1.0
Moderate	5,558	4,097	1,428	1.11 (1.04-1.18)	1.09 (1.02-1.18)	1.17 (1.02-1.35)
High/Severe	1,028	1,071	380	1.56 (1.4-1.75)	1.61 (1.41-1.82)	1.47 (1.17-1.85)

© 2022 Reddin C et al. *JAMA Network Open*.

**Stressful Life Events**						
None	9,285	6,614	2,130	1.0	1.0	1.0
1 Event	2,712	2,320	552	1.17 (1.09-1.25)	1.2 (1.11-1.3)	1.03 (0.88-1.21)
>=2 Events	1,533	1,422	372	1.31 (1.19-1.43)	1.33 (1.2-1.47)	1.27 (1.04-1.56)
[1]Adjusted for age (and matched for sex and centre), occupation, wealth index and education (conditional); [2]Included only those working						

© 2022 Reddin C et al. *JAMA Network Open*.

eTable 3. Association Between Stressful Life Events and Stroke Types (Conditional Logistic Regression)

Risk Factors	Multivariate analysis[1]		
	**All stroke**	**Ischemic stroke**	**ICH**
**Divorce**			
No	1.0	1.0	1.0
Yes	1.33 (1.07-1.66)	1.51 (1.21-1.89)	1.51 (1.21-1.89)
**Loss of job**			
No	1.0	1.0	1.0
Yes	1.2 (1.04-1.38)	1.29 (1.11-1.49)	1.29 (1.11-1.49)
**Business failure or loss of crop**			
No	1.0	1.0	1.0
Yes	0.94 (0.81-1.08)	0.91 (0.78-1.07)	0.91 (0.78-1.07)
**Violence**			
No	1.0	1.0	1.0
Yes	1.26 (1.03-1.54)	1.39 (1.13-1.72)	1.39 (1.13-1.72)
**Major intra-family conflict**			
No	1.0	1.0	1.0
Yes	1.77 (1.60-1.96)	2.21 (1.99-2.46)	2.21 (1.99-2.46)
**Death of a family member**			
No	1.0	1.0	1.0
Yes	0.93 (0.86-1)	0.9 (0.83-0.98)	0.9 (0.83-0.98)
**Death of a spouse**			
No	1.0	1.0	1.0
Yes	1.35 (1.11-1.63)	1.51 (1.23-1.85)	1.51 (1.23-1.85)
**Other Stress**			
No	1.0	1.0	1.0
Yes	1.56 (1.38-1.77)	1.87 (1.64-2.13)	1.87 (1.64-2.13)

[1]Adjusted for age, occupation, wealth index and matched for age, sex, and centre.

© 2022 Reddin C et al. *JAMA Network Open*.

eTable 4. Composite of Stress and Control Prevalence and Unconditional Logistic Regression

		Prevalence			All stroke	P for interaction
Home Stress	**Life Control**	**Controls N**	**Ischemic N**	**ICH N**	**Odds Ratio**	
Never	High control	2,035	1,246	265	1.0	<0.001
	Neutral control	1,352	969	310	1.23 (1.11-1.37)	
	Low control	1,841	1,208	369	1.12 (1.02-1.24)	
Some of the time	High control	2,524	1,647	327	1.08 (0.98-1.18)	
	Neutral control	1,973	1,706	645	1.62 (1.47-1.78)	
	Low control	2,469	1,994	631	1.51 (1.38-1.66)	
Several Periods/Permanent	High control	374	359	86	1.69 (1.44-1.98)	
	Neutral control	294	299	99	1.89 (1.59-2.24)	
	Low control	629	799	232	2.40 (2.11-2.72)	
Female						
Home Stress	**Life Control**	**Controls**	**Ischemic**	**ICH**	**Odds Ratio**	**P for interaction**
Never	High control	694	413	102	1.0	0.065
	Neutral control	534	357	123	1.15 (0.97-1.37)	
	Low control	685	449	155	1.15 (0.97-1.36)	
Some of the time	High control	1,027	647	123	1.04 (0.89-1.21)	
	Neutral control	830	714	255	1.5 (1.28-1.75)	
	Low control	997	826	232	1.46 (1.25-1.7)	
Several Periods/Permanent	High control	186	166	46	1.59 (1.26-2.01)	
	Neutral control	159	151	39	1.68 (1.31-2.15)	
	Low control	331	393	113	2.16 (1.79-2.6)	
Male						
Home Stress	**Life Control**	**Controls**	**Ischemic**	**ICH**	**Odds Ratio**	**P for interaction**
Never	High control	1,341	833	163	1.0	0.02
	Neutral control	818	612	187	1.25 (1.1-1.43)	
	Low control	1,156	759	214	1.09 (0.96-1.23)	
Some of the time	High control	1,497	1,000	204	1.09 (0.98-1.23)	
	Neutral control	1,143	992	390	1.61 (1.43-1.81)	
	Low control	1,472	1,168	399	1.46 (1.31-1.64)	
Several Periods/Permanent	High control	188	193	40	1.74 (1.41-2.15)	
	Neutral control	135	148	60	2.06 (1.62-2.61)	
	Low control	298	406	119	2.54 (2.14-3.01)	
Work Stress	**Work Control**	**Controls**	**Ischemic**	**ICH**	**Odds Ratio**	**P for interaction**
Never	High control	1,067	640	216	1.0	0.008
	Neutral control	269	147	53	1.05 (0.85-1.31)	
	Low control	210	92	36	0.85 (0.66-1.09)	
Some of the time	High control	1,907	1,108	419	1.16 (1.03-1.31)	

© 2022 Reddin C et al. *JAMA Network Open*.

	Neutral control	781	539	231	1.45 (1.25-1.68)	
	Low control	468	292	98	1.17 (0.99-1.4)	
Several Periods/Permanent	High control	504	499	164	2.20 (1.88-2.58)	
	Neutral control	191	166	71	1.97 (1.57-2.47)	
	Low control	175	223	68	2.70 (2.16-3.37)	
Female						
Work Stress	**Work Control**	**Controls**	**Ischemic**	**ICH**	**Odds Ratio**	**P for interaction**
Never	High control	274	153	75	1.0	0.77
	Neutral control	87	42	15	1.1 (0.72-1.67)	
	Low control	65	22	13	0.87 (0.54-1.4)	
Some of the time	High control	429	234	89	1.24 (0.96-1.6)	
	Neutral control	197	107	48	1.33 (0.97-1.82)	
	Low control	137	83	25	1.16 (0.82-1.63)	
Several Periods/Permanent	High control	131	107	38	2.25 (1.62-3.14)	
	Neutral control	49	30	12	1.68 (1.03-2.73)	
	Low control	60	50	12	1.93 (1.26-2.98)	
Male						
Work Stress	**Work Control**	**Controls**	**Ischemic**	**ICH**	**Odds Ratio**	**P for interaction**
Never	High control	793	487	141	1.0	0.005
	Neutral control	182	105	38	1.01 (0.79-1.3)	
	Low control	145	70	23	0.81 (0.61-1.08)	
Some of the time	High control	1,478	874	330	1.13 (0.98-1.29)	
	Neutral control	584	432	183	1.43 (1.21-1.69)	
	Low control	331	209	73	1.14 (0.94-1.39)	
Several Periods/Permanent	High control	373	392	126	2.15 (1.79-2.57)	
	Neutral control	142	136	59	2.02 (1.57-2.6)	
	Low control	115	173	56	3 (2.31-3.89)	

© 2022 Reddin C et al. *JAMA Network Open*.

eTable 5. Association of Work and Home Stress With All Stroke Stratified by Control

Risk Factors			P for Interaction
**Work Stress**	**High Work Control**	**Low Work Control**	
Never	1.0	1.0	0.009
Some of the time	1.12 (0.98-1.28)	1.99 (1.66-2.39)	
Several Periods/Permanent	1.75 (1.47-2.1)	4.51 (3.54-5.75)	
**Home Stress**	**High Life Control**	**Low Life Control**	
Never	1.0	1.0	0.009
Some of the time	1.06 (0.96-1.17)	1.32 (1.24-1.41)	
Several Periods/Permanent	1.17 (1-1.36)	2.35 (2.12-2.6)	

eTable 6. Several Periods of General Stress or Permanent General Stress Prevalence and Conditional Logistic Regression by Region

	Prevalence			Odds Ratio (95% CI)			P for interaction
Region	**Controls (N)**	**Ischemic (N)**	**ICH (N)**	**All stroke**	**Ischaemic stroke**	**ICH**	
Western Europe/ North America	435	495	40	1.58 (1.28-1.96)	1.56 (1.25-1.94)	2.8 (1.04-7.55)	<0.001
Eastern/central Europe/Middle East	282	321	42	2.05 (1.58-2.66)	1.95 (1.48-2.57)	3.43 (1.27-9.27)	
Africa	248	179	86	1.1 (0.78-1.55)	0.86 (0.57-1.31)	2 (1.04-3.84)	
South Asia	311	427	172	2.33 (1.84-2.95)	2.2 (1.67-2.9)	2.68 (1.69-4.24)	
China	201	256	108	2.58 (2.04-3.27)	2.54 (1.94-3.34)	2.75 (1.68-4.51)	
South East Asia	223	149	92	2.21 (1.48-3.32)	2.28 (1.43-3.62)	1.28 (0.47-3.46)	
South America	244	274	93	2.67 (2.03-3.53)	2.41 (1.77-3.29)	5.22 (2.54-10.71)	

© 2022 Reddin C et al. *JAMA Network Open*.

eTable 7. Psychosocial Stress Prevalence and Conditional Logistic Regression by Sex

	Prevalence: Men			All stroke	Prevalence: Women			All stroke	P for interaction
**Risk Factors**	**Controls (N)**	**Ischemic (N)**	**ICH (N)**	**Odds Ratio (95% CI)**	**Controls (N)**	**Ischemic (N)**	**ICH (N)**	**Odds Ratio (95% CI)**	
**Martial Separation or Divorce:**									0.26
No	7,915	6,042	1,808	1.0	5,349	4,114	1,213	1.0	
Yes	95	110	23	1.47 (1.12-1.92)	86	85	9	1.15 (0.85-1.56)	
**Loss of job or retirement:**									0.73
No	7,625	5,812	1,736	1.0	5,282	4,068	1,195	1.0	
Yes	386	343	96	1.17 (1.01-1.36)	153	132	27	1.06 (0.82-1.36)	
**Business failure or loss of crop:**									0.2
No	7,708	5,913	1,753	1.0	5,259	4,083	1,192	1.0	
Yes	303	243	79	1.09 (0.92-1.28)	176	117	30	0.83 (0.66-1.05)	
**Violence:**									0.49
No	7,883	6,033	1,783	1.0	5,352	4,122	1,200	1.0	
Yes	127	123	49	1.38 (1.09-1.74)	83	78	22	1.23 (0.91-1.67)	
**Major intra-family conflict:**									0.02
No	7,585	5,596	1,642	1.0	4,956	3,647	1,089	1.0	
Yes	425	559	190	1.96 (1.72-2.23)	478	553	133	1.64 (1.43-1.88)	
**Personal Injury:**									0.0052
No	7,314	5,695	1,715	1.0	4,882	3,712	1,142	1.0	

© 2022 Reddin C et al. *JAMA Network Open*.

Yes	693	461	117	0.81 (0.72-0.91)	551	488	80	1.04 (0.91-1.19)	
**Death of a family member:**									0.09
No	6,899	5,365	1,604	1.0	4,483	3,400	1,055	1.0	
Yes	1,108	791	228	0.9 (0.81-0.99)	950	800	167	1.04 (0.93-1.16)	
**Death of a spouse:**									0.97
No	7,919	6,060	1,802	1.0	5,290	4,026	1,189	1.0	
Yes	90	93	30	1.46 (1.1-1.94)	143	174	33	1.55 (1.23-1.96)	
**Other stress:**									0.008
No	7,707	5,812	1,722	1.0	5,135	3,905	1,159	1.0	
Yes	297	343	109	1.69 (1.44-1.98)	294	295	63	1.27 (1.07-1.51)	
**Work Stress:**									0.17
Never	1,115	665	205	1.0	424	220	103	1.0	
Some of the time	2,373	1,525	602	1.35 (1.17-1.56)	763	428	165	1.33 (1-1.76)	
Several Periods/Permanent	628	706	247	2.43 (2.02-2.92)	241	189	66	1.68 (1.18-2.38)	
**Home Stress:**									0.18
Never	3,300	2,211	575	1.0	1,916	1,236	388	1.0	
Some of the time	4,091	3,175	1,007	1.35 (1.25-1.46)	2,842	2,217	618	1.28 (1.16-1.41)	
Several Periods/Permanent	615	750	222	2.13 (1.89-2.41)	671	719	201	1.85 (1.62-2.12)	
**Financial Stress:**									0.45
Little/none	4,106	3,030	690	1.0	2,824	2,114	512	1.0	
Moderate	3,303	2,478	873	1.21 (1.12-1.3)	2,194	1,619	555	1.13 (1.03-1.23)	
High/Severe	602	630	241	1.87 (1.64-2.13)	416	441	139	1.75 (1.49-2.05)	

© 2022 Reddin C et al. *JAMA Network Open*.

eTable 8. Psychosocial Stress Prevalence and Conditional Logistic Regression by Age

	Prevalence: <45			All Stroke	Prevalence: 45 to 65			All stroke	Prevalence: >65			All Stroke	
Risk Factors	**Controls (N)**	**Ischemic (N)**	**ICH (N)**	**Odds Ratio (95% CI)**	**Controls (N)**	**Ischemic (N)**	**ICH (N)**	**Odds Ratio (95% CI)**	**Controls (N)**	**Ischemic (N)**	**ICH (N)**	**Odds Ratio (95% CI)**	**P for interaction**
Martial Separation or Divorce:													
No	1,545	1,081	448	1.0	5,676	4,123	1,500	1.0	6,043	4,952	1,073	1.0	0.26
Yes	31	41	10	1.81 (1.11-2.94)	96	96	17	1.3 (0.97-1.75)	54	58	5	1.25 (0.81-1.93)	
Loss of job or retirement:													
No	1,518	1,068	435	1.0	5,518	3,990	1,452	1.0	5,871	4,822	1,044	1.0	0.7
Yes	59	54	23	1.38 (0.93-2.05)	254	231	65	1.26 (1.04-1.53)	226	190	35	1.15 (0.9-1.46)	
Business failure or loss of crop:													
No	1,502	1,061	430	1.0	5,526	4,031	1,460	1.0	5,939	4,904	1,055	1.0	0.05
Yes	75	61	28	1.13 (0.79-1.61)	246	190	57	0.95 (0.78-1.16)	158	109	24	0.88 (0.67-1.16)	
Violence:													
No	1,544	1,078	440	1.0	5,662	4,128	1,480	1.0	6,029	4,949	1,063	1.0	0.11
Yes	32	44	18	2.07 (1.27-3.37)	110	93	37	1.06 (0.8-1.39)	68	64	16	1.21 (0.82-1.78)	
Major intra-family conflict:													
No	1,465	995	405	1.0	5,393	3,786	1,361	1.0	5,683	4,462	965	1.0	0.53
Yes	111	127	53	1.95 (1.45-2.6)	379	434	156	1.77 (1.52-2.06)	413	551	114	1.8 (1.54-2.12)	
Major personal injury/illness:													
No	1,453	1,022	427	1.0	5,291	3,893	1,436	1.0	5,452	4,492	994	1.0	0.096
Yes	123	100	31	1.01 (0.75-1.35)	480	328	81	0.75 (0.65-0.88)	641	521	85	0.82 (0.71-0.95)	
Death of a family member:													
No	1,327	929	399	1.0	4,935	3,655	1,317	1.0	5,120	4,181	943	1.0	0.12
Yes	249	193	59	1.05 (0.84-1.32)	836	566	200	0.86 (0.76-0.98)	973	832	136	0.98 (0.87-1.1)	
Death of a spouse:													

© 2022 Reddin C et al. *JAMA Network Open*.

No	1,574	1,115	454	1.0	5,709	4,156	1,493	1.0	5,926	4,815	1,044	1.0	0.059
Yes	3	7	4	4.11 (1.11-15.27)	62	63	24	1.28 (0.89-1.84)	168	197	35	1.25 (0.98-1.6)	
Other Stress:													
No	1,488	1,017	421	1.0	5,529	3,952	1,429	1.0	5,825	4,748	1,031	1.0	0.00056
Yes	87	105	37	2.25 (1.59-3.17)	240	269	87	1.77 (1.46-2.15)	264	264	48	1.32 (1.08-1.61)	
Work Stress:													
Never	282	138	54	1.0	930	546	197	1.0	327	201	57	1.0	0.59
Some of the time	752	450	195	1.59 (1.21-2.1)	1,908	1,215	481	1.53 (1.28-1.82)	476	288	91	1.03 (0.59-1.8)	
Several Periods/Permanent	253	256	94	3.21 (2.29-4.5)	536	552	192	2.64 (2.10-3.32)	80	87	27	2.26 (1.03-4.97)	
Home Stress:													
Never	514	302	116	1.0	2,167	1,320	463	1.0	2,535	1,825	384	1.0	0.4
Some of the time	909	620	256	1.32 (1.07-1.63)	3,007	2,238	809	1.32 (1.2-1.46)	3,017	2,534	560	1.32 (1.2-1.46)	
Several Periods/Permanent	155	198	75	2.49 (1.86-3.33)	598	643	221	1.97 (1.71-2.28)	533	628	127	1.84 (1.58-2.15)	
Financial Stress:													
Little/none	590	409	120	1.0	2,818	1,795	558	1.0	3,522	2,940	524	1.0	<0.001
Moderate	801	522	244	1.05 (0.87-1.26)	2,463	1,896	741	1.3 (1.18-1.43)	2,233	1,679	443	0.98 (0.88-1.08)	
High/Severe	187	189	83	1.43 (1.08-1.91)	490	512	193	1.85 (1.58-2.17)	341	370	104	1.31 (1.08-1.59)	

© 2022 Reddin C et al. *JAMA Network Open*.

eTable 9. Association of Stress Domains and Stroke (Sensitivity Analysis)

Risk Factors	Odds Ratio (95% CI)		
	**Multivariate analysis[1]**		
	**All stroke**	**Ischaemic stroke**	**ICH**
**General Stress[1]**			
Never	1.0	1.0	1.0
Some of the time	1.22 (1.14-1.29)	1.2 (1.12-1.29)	1.25 (1.09-1.43)
Several Periods/Permanent	1.93 (1.77-2.1)	1.8 (1.64-1.98)	2.39 (1.99-2.89)
**Work Stress[2]**			
Never	1.0	1.0	1.0
Some of the time	1.21 (1.1-1.33)	1.23 (1.09-1.4)	1.95 (1.47-2.61)
Several Periods/Permanent	2.23 (1.97-2.52)	2.07 (1.76-2.44)	5.2 (3.48-7.77)
**Home Stress[3]**			
Never	1.0	1.0	1.0
Some of the time	1.22 (1.15-1.29)	1.2 (1.12-1.28)	1.25 (1.1-1.42)
Several Periods/Permanent	1.69 (1.55-1.86)	1.62 (1.47-1.8)	1.94 (1.59-2.38)
**Composite of Home and Work Stress[4]**			
Neither	1.0	1.0	1.0
Mild work or home stress	1.25 (1.1-1.42)	1.3 (1.1-1.54)	1.66 (1.25-2.2)
Both mild	1.28 (1.14-1.44)	1.32 (1.13-1.54)	1.71 (1.33-2.19)
One severe	2.12 (1.84-2.44)	2.04 (1.7-2.45)	3.44 (2.51-4.75)
Both severe	2.74 (2.24-3.35)	2.38 (1.83-3.11)	5.46 (3.37-9.08)
**Financial Stress[5]**			
Little/none	1.0	1.0	1.0
Moderate	0.94 (0.88-0.99)	0.95 (0.89-1.01)	0.95 (0.83-1.07)
High/Severe	1.12 (1.01-1.24)	1.16 (1.04-1.31)	1.03 (0.84-1.27)

[1]Adjusted for age (and matched for sex and centre), occupation, wealth index, education and financial stress ; [2] Adjusted for age (and matched for sex and centre), occupation, wealth index and education, home stress and financial stress. Included only those working ; [3] Adjusted for age (and matched for sex and centre), occupation, wealth index, education, work stress and financial stress ; [4] Adjusted for age (and matched for sex and centre), occupation, wealth index and education, and financial stress. Included only those working; [5]Adjusted for age (and matched for sex and centre), occupation, wealth index, education, work stress and home stress.

© 2022 Reddin C et al. *JAMA Network Open*.

eFigure. Multivariate Logistic Regression Models (Conditional)

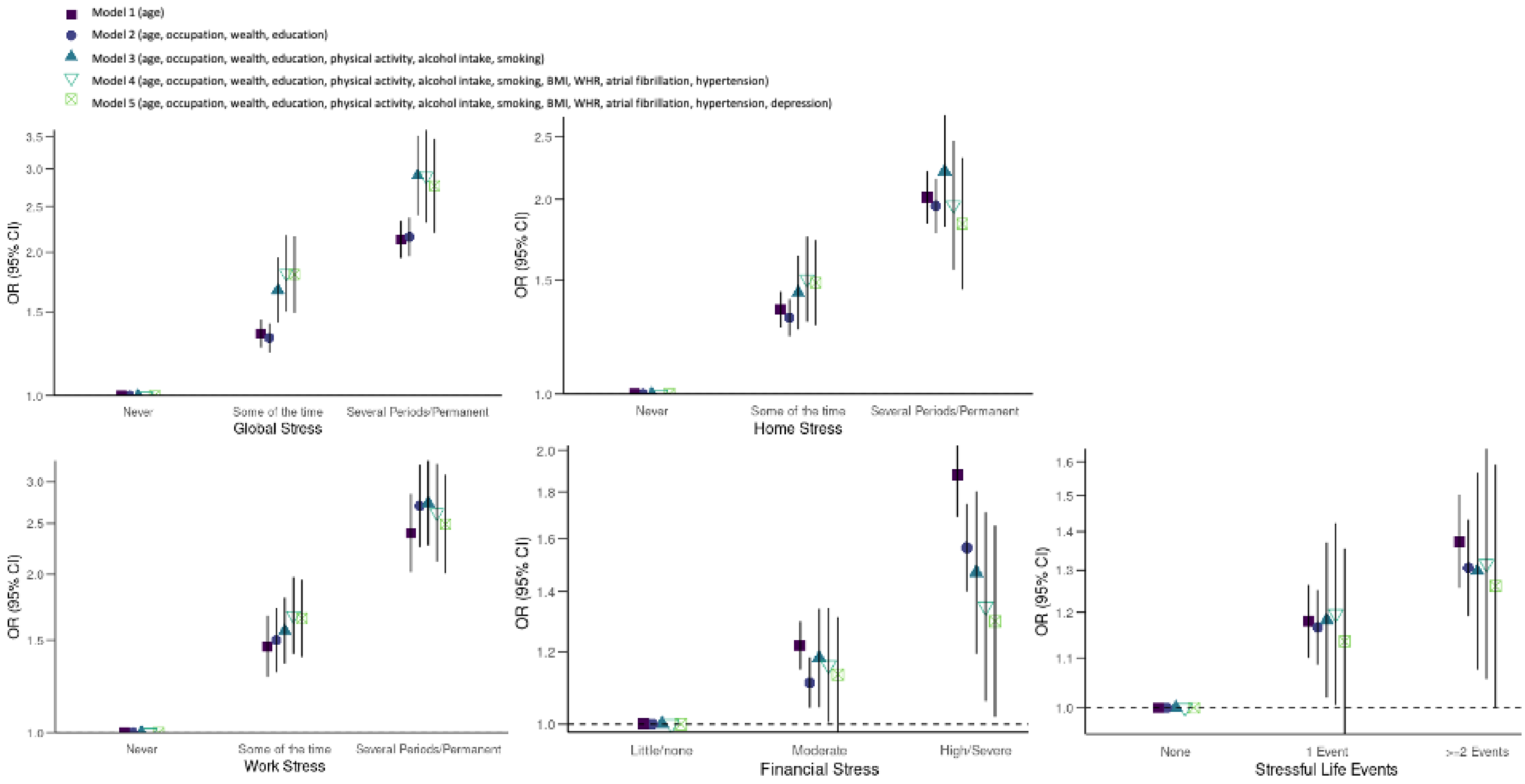

© 2022 Reddin C et al. *JAMA Network Open*.

eAppendix. INTERSTROKE Study Site Staff

Project Office Staff, National Coordinators, Investigators and Key Staff Project office staff (Population Health Research Institute, Hamilton Health Sciences and McMaster University, Hamilton, Canada): Coordination and Data Management: M. O'Donnell, S. Yusuf (Principal Investigators) S. Rangarajan (Project Manager); P. Rao-Melacini, X. (Michelle) Zhang, S. Islam, C. Kabali, A. Casanova (Statisticians); S.L. Chin, J. DeJesus (Study Coordinator), M. Dehghan (Nutritionist), S. Agapay.

Core Laboratories: M. McQueen, K. Hall, J. Keys (Hamilton), X. Wang (Beijing, China), A. Devanath (Bangalore, India), R. Gupta, D. Prabhakaran (New Delhi, India)

ARGENTINA: R. Diaz*, P. Schygiel, M. Garrote, M.A. Rodriguez, A. Caccavo, R.G. Duran, L. Sposato, J. Molinos, P. Valdez, C.M. Cedrolla, P.G. Nofal, M.F. Huerta, P.M Desmery, M.C. Zurru, B. Della Vedova;

AUSTRALIA: J. Varigos*, G. Hankey*, T. Kraemer, P. Gates, C. Bladin, G. Herkes;

BRAZIL: A. Avezum*, M.P. Pereira, L. Minuzzo, L. Oliveira, M. Teixeira, H. Reis, A. Carvalho, S. Ouriques Martins, J.J. Carvalho, O. Gebara, C. Minelli, D.C. Oliveira, A.C. Sobral Sousa, A.C. Ferraz de Almeida, M.E. Hernandez, M. Friedrich, D.M. Mota, L.E. Ritt, D. Correa Vila Nova;

CANADA: M. O'Donnell*, S. Yusuf, P. Teal, D. Gladstone, A. Shuaib, F. Silver, D. Dowlatshahi, J. DeJesus, S. Rangarajan, S. Agapay, S.L. Chin;

CHILE: F. Lanas*, D. Carcamo, C. Santibañez, E. Garces;

CHINA: L.S. Liu*, H.Y. Zhang*, H.P. Fang, M.F. Lian, F. Shen, F.X Luo, X.X. Wen, Z.Q. Xu, Z.Z Liu, W. Yan, J.F. Yu, W.K. Wang, L.H. Liu, Y.H. Sun, L.C. Zhou, Z.F. Zhang, J. Lv, C.S. Zhang, G. Chen, H.L. Wang, Y. Chen, H. Zheng, J.J. Huang, W.Z. Li, L.J. Wang, J.X. Shi, C.Y. Hu, H.F. Song, R.Y. Ji, D.L. Wang, L.H. Meng, Q.W. Meng, L.J. Duan, H.F. Liu, Y.C. Luo, Q.Y. Zhang, Y.B. Wu, C.R. Wang, J.G. Zhao, S.G. Liu, C.L. Shi, X.Y. Wang;

COLOMBIA: P. Lopez-Jaramillo*, A. Martinez, G. Sanchez-Vallejo, D.I. Molina, T. Espinosa, H. Garcia Lozada, D. Gomez-Arbelaez, P.A. Camacho;

CROATIA: Z. Rumboldt*, I. Lusic;

DENMARK: H. K. Iversen*, T. Truelsen, C. Back, M. M. Pedersen;

ECUADOR: E. Peñaherrera*, Y.C. Duarte, S. Cevallos, D. Tettamanti, S. Caceres;

GERMANY: H.C. Diener*, C.Weimar, A. Grau, J. Rother, M. Ritter, T. Back, Y. Winter;

INDIA: P. Pais*, D. Xavier* (co-Principal Investigator for INTERSTROKE), A. Sigamani, N. Mathur, P. Rahul, A. Murali, A.K. Roy, G.R.K. Sarma, T. Matthew, G. Kusumkar, K.A. Salam, U. Karadan, L. Achambat, Y. Singh, J.D. Pandian, R. Verma, V. Atam, A. Agarwal, N. Chidambaram, R. Umarani, S. Ghanta, G.K. Babu, G. Sathyanarayana, G. Sarada, S. Navya Vani, R. Sundararajan, S.S. Sivakumar, R.S. Wadia, S. Bandishti, R. Gupta, R.R. Agarwal, I. Mohan, S. Joshi, S. Kulkarni, S. Partha Saradhi , P. Joshi, M. Pandharipande, N. Badnerkar, R. Joshi, S.P. Kalantri, S. 18 Somkumar, S. Chauhan, H. Singh, S. Varma, H. Singh, G.K. Sidhu, R. Singh, K.L. Bansal, A. Bharani, S. Pagare, A. Chouhan, B.N. Mahanta, T.G. Mahanta, G. Rajkonwar, S.K. Diwan, S.N. Mahajan, P. Shaikh, H.R. Devendrappa, B. K. Agrawal, A. Agrawal, D. Khurana, S. Thakur, V. Jain;

IRAN: S. Oveisgharan*, A. Bahonar, R. Kelishadi, A. Hossienzadeh, M. Raeisidehkordi, H. Akhavan;

IRELAND: M. O'Donnell*, T. Walsh;

KUWAIT: O. Albaker*;

MALAYSIA: K. Yusoff*, A. Chandramouli, S. Shahadan, Z. Ibrahim, A. Husin;

© 2022 Reddin C et al. *JAMA Network Open*.

MOZAMBIQUE: A. Damasceno*, V. Lobo, S. Loureiro, V.A. Govo;

NIGERIA: O.S. Ogah*, A. Ogunniyi*, R.O. Akinyemi, M.O. Owolabi, M.U. Sani, L.F. Owolabi;

PAKISTAN: R. Iqbal*, M. Wasay, A. Raza; PERU: G.G. Malaga*, M. LazoPorras, J.D. Loza-Herrera , A. Acuña-Villaorduña, D. Cardenas-Montero;

PHILIPPINES: A. Dans*, E. Collantes, D. Morales, A. Roxas, M.V.C. Villarruz-Sulit;

POLAND: A. Czlonkowska*, D. Ryglewicz*, M. Skowronska, M. Restel, A. Bochynska, K. Chwojnicki, M. Kubach, A. Stowik, M. Wnuk;

RUSSIA: N. Pogosova*, A. Ausheva, A. Karpova, V. Pshenichnikova, A. Vertkin; A. Kursakov, S. Boytsov;

SAUDI ARABIA: F. Al-Hussain*;

SOUTH AFRICA: L. DeVilliers*, D. Magazi, B. Mayosi;

SUDAN: A.S.A. Elsayed*, A. Bukhari, Z. Sawaraldahab, H. Hamad, M. ElTaher, A. Abdelhameed, M. Alawad, D. Alkabashi, H. Alsir;

SWEDEN: A. Rosengren*, M. Andreasson, J. Kembro Johansson, B. Cederin, C. Schander, A.C. Elgasen, E. Bertholds, K. Boström Bengtsson;

THAILAND: Y. Nilanont*, S. Nidhinandana*, P. Tatsanavivat, N. Prayoonwiwat, N. Poungvarin, N.C. Suwanwela, S. Tiamkao, R. Tulyapornchote, S. Boonyakarnkul, S. Hanchaiphiboolkul, S. Muengtaweepongsa, K. Watcharasaksilp, P. Sathirapanya, P. Pleumpanupat;

© 2022 Reddin C et al. *JAMA Network Open*.

TURKEY: A. Oguz*, A.A. Akalin, O.T. Caklili, N. Isik, B. Caliskan, B. Sanlisoy, E. Balkuv, H. Tireli, V. Yayla, M. Cabalar, A. Culha, S. Senadim, B. Arpaci, C. Dayan, T. Argun, S. Yilmaz, S. Celiker, A. Kocer, T. Asil, G. Eryigit,

UGANDA: C. Mondo*, J. Kayima, M. Nakisige, S. Kitoleeko;

UNITED ARAB EMIRATES: A.M. Yusufali*, B.J. Zuberi, H.Z. Mirza, A.A. Saleh, J.M. BinAdi, F Hussain;

UNITED KINGDOM: P. Langhorne*, K. Muir, M. Walters, C. McAlpine, S. Ghosh, M. Barber, N. Hughes, M.J. MacLeod, S. Ghosh, A. Doney, S. Johnston, P. Mudd, T. Black, P. Murphy, D. Jenkinson, D. Kelly, R. Whiting, D. Dutta, L. Shaw, C. Mcfarlane, E. Ronald, K McBurnie.

*National Coordinator
