# Supplementary material for: Association of Psychosocial Stress With Risk of Acute Stroke
Source: JAMA Netw Open. 2022 Dec 9;5(12):e2244836. doi: 10.1001/jamanetworkopen.2022.44836 (PMC9856236; doi:10.1001/jamanetworkopen.2022.44836)
Supplement: Supplement 2. — Nonauthor Collaborators [file jamanetwopen-e2244836-s002.pdf]

Supplemental Online Content: Nonauthor Collaborators
*First name, last name, and suffix (if applicable) are required and will appear in PubMed.

*Group Name(s): INTERSTROKE investigators							
***First Name and Middle Initial(s)**	***Last Name**	***Suffix (eg, Jr, III)**	Academic Degrees	Institution	Location (city, state/province, country)	Role or Contribution, eg, chair, principal investigator	Group (if more than 1 Group listed in the byline) and/or Subgroup (eg, Steering Committee)
Sumathy	Rangaran						
Purnima	Rao-Melacini						
Xiaohe	Zhang						
Shofiqul	Islam						
Conrad	Kabali						
Amparo	Cassanova						
Siu Lim	Chin						
Jane	DeJesus						
Mahshid	Dehghan						
Steven	Agapay						
Rafael	Diaz						
John	Varigos						
Alvaro	Avezum						
Lisheng	Liu						
Hongye	Zhang						
Patricio	Lopez-Jaramillo						
Zvonko	Rumboldt						
Ernesto	Peñaherrera						
Hans-Christopher	Diener						
Prem	Pais						
Osamah	Albaker						
Khalid	Yusoff						
Albertino	Damasceno						
Okechukwu	Ogah						
Romaina	Iqbal						
Antonio	Dans						
Danuta	Ryglewicz						
Linda	De Villiers						
Ahmed	Elsayed						

Supplemental Online Content: Nonauthor Collaborators
*First name, last name, and suffix (if applicable) are required and will appear in PubMed.

***First Name and Middle Initial(s)**	***Last Name**	***Suffix (eg, Jr, III)**	Academic Degrees	Institution	Location (city, state/province, country)	Role or Contribution, eg, chair, principal investigator	Group (if more than 1 Group listed in the byline) and/or Subgroup (eg, Steering Committee)
Yongchai	Nilanont						
Samart	Nidhinandana						
Charles	Mondo						
Alfzalhussein	Yusufali						
