# Supplementary material for: Association of Psychosocial Stress With Risk of Acute Stroke
Source: JAMA Netw Open. 2022 Dec 9;5(12):e2244836. doi: 10.1001/jamanetworkopen.2022.44836 (PMC9856236; doi:10.1001/jamanetworkopen.2022.44836)
Supplement: Supplement 3. — Data Sharing Statement [file jamanetwopen-e2244836-s003.pdf]

Data Sharing Statement

Reddin. Association of Psychosocial Stress With Risk of Acute Stroke. *JAMA Netw Open*. Published December 09, 2022. doi:10.1001/jamanetworkopen.2022.44836

Data
Data available: No
